# Blocking EGR1/TGF-β1 and CD44s/STAT3 Crosstalk Inhibits Peritoneal Metastasis of Gastric Cancer

**DOI:** 10.7150/ijbs.90598

**Published:** 2024-01-27

**Authors:** Yangbing Jin, Chao Wang, Benyan Zhang, Ying Sun, Jun Ji, Qu Cai, Jinling Jiang, Zhihao Zhang, Liqin Zhao, Beiqin Yu, Jun Zhang

**Affiliations:** 1 Department of Oncology, Ruijin Hospital, Shanghai Jiao Tong University School of Medicine, Shanghai, 200025, China.; 2 Department of General Surgery, Shanghai Key Laboratory of Gastric Neoplasms, Shanghai Institute of Digestive Surgery, Ruijin Hospital, Shanghai Jiao Tong University School of Medicine, Shanghai, 200025, China.; 3 Department of Pathology, Ruijin Hospital, Shanghai Jiao Tong University School of Medicine, Shanghai, 200025, China.; 4 Clinical Research and Development, Jiangsu Hengrui Pharmaceuticals Co. Ltd, Shanghai, 201203, China.; 5 Department of Oncology, Wuxi Branch of Ruijin Hospital, Shanghai Jiao Tong University School of Medicine, No 197 Zhixian Road, Xinwu District, Wuxi, 214028, China.

**Keywords:** gastric cancer, peritoneal metastasis, EGR1, TGF-β1, SHR-1701

## Abstract

Peritoneal metastasis (PM) continues to limit the clinical efficacy of gastric cancer (GC). Early growth response 1 (EGR1) plays an important role in tumor cell proliferation, angiogenesis and invasion. However, the role of EGR1 derived from the tumor microenvironment in reshaping the phenotypes of GC cells and its specific molecular mechanisms in increasing the potential for PM are still unclear. In this study, we reported that EGR1 was significantly up-regulated in mesothelial cells from GC peritoneal metastases, leading to enhanced epithelial-mesenchymal transformation (EMT) and stemness phenotypes of GC cells under co-culture conditions. These phenotypes were achieved through the transcription and secretion of TGF-β1 by EGR1 in mesothelial cells, which could regulate the expression and internalization of CD44s. After being internalized into the cytoplasm, CD44s interacted with STAT3 to promote STAT3 phosphorylation and activation, and induced EMT and stemness gene transcription, thus positively regulating the metastasis of GC cells. Moreover, TGF-β1 secretion in the PM microenvironment was significantly increased compared with the matched primary tumor. The blocking effect of SHR-1701 on TGF-β1 was verified by inhibiting peritoneal metastases in xenografts. Collectively, the interplay of EGR1/TGF-β1/CD44s/STAT3 signaling between mesothelial cells and GC cells induces EMT and stemness phenotypes, offering potential as a therapeutic target for PM of GC.

## Introduction

Peritoneal metastasis (PM) remains a major hurdle to long-lasting survival of patients with gastric cancer (GC). This can be partly explained by the peritoneal-plasma barrier and poor cancer tissue vascularity in PM 1-3. Researches in cancer biology give priority to examining the interactions of the peritoneal microenvironment and malignant ascites with tumor cells, because the reciprocal crosstalk of these interactions could be identified as potential therapeutic targets. Therefore, clarifying the characteristic molecular mechanisms in PM of GC and exploring alterations of the cell molecular events that take part in the development of PM will provide important foundations for clinical prevention and treatment in PM of GC.

A previous study from our group described a comprehensive gene spectrum of advanced GC through whole-genome and transcriptome sequencing analysis of primary GC, matched PM and normal gastric mucosa. The work identified and reported a group of aberrantly expressed and mutated genes in primary GC and PM, and found that homeobox a11 (HOXA11) expression in tumor tissues was significantly up-regulated compared to the normal tissues 4, 5. Subsequent *in vitro* and *in vivo* studies from our group indicated that GC cells could act as "seeds" to induce alterations in the "soil" microenvironment via HOXA11, and cause human peritoneal mesothelial cells (HPMCs) to undergo mesenchymal transition (MMT). Transcriptome sequencing revealed the up-regulation of 12 genes, including EGR1, in HPMCs after co-culture with HOXA11-highly expressed GC cells (NCI-N87 and SGC-7901) 6.

Early growth response 1 (EGR1) is a critical transcription factor and is known to be involved in various pathophysiological processes, including cell proliferation, migration, differentiation and fibrosis by increasing the transcription of cytokines, chemokines associated target genes. It plays a role in fibrosis in liver, lung, skin and oral mucosa. Additionally, it contributes to radiotherapy-associated interstitial fibrosis in breast cancer by regulating α-SMA, collage, TGF-β1 expression and promoting the transition of fibroblasts into myofibroblasts 7-11. Furthermore, in glioblastoma stem cells, EGR1 positively regulates stemness-related genes at the transcriptional levels, such as Shh, Gli1, Gli2 and PDGFA 12. In radiation-induced breast cancer stem cells, EGR1 remodels the tumor stroma to promote tumor metastasis by inducing protease connexin-1 (PN-1) upregulation 13.

Mesothelial cells are plastic non-terminally differentiated cells. Now it is confirmed that they have the ability to transform into a variety of terminal interstitial cells, such as antigen-presenting cancer-associated fibroblasts (CAFap) or macrophages, thereby participating in the process of tumor microenvironment remodeling 14, 15. Despite the low expression of EGR1 in mesothelial cells under normal conditions, a significant increase in EGR1 can be observed under inflammatory and tumor conditions. Therefore, we hypothesized that EGR1-overexpressing mesothelial cells may alter the microenvironment through cell-cell interaction, thereby influencing the characteristics of GC cells and promoting PM. Hence, we conducted the present study to test this hypothesis.

## Methods

### Clinical sample collection

Clinical samples of eight cases of GC PM tissues and their matched paracancerous tissues were obtained from Ruijin Hospital, Shanghai Jiao Tong University School of Medicine for multiple immunofluorescence staining (EGR1, HBME1, HOXA11, CD44). Another 12 GC samples and their matched PM were collected for analysis at the transcriptional level. These 12 patients underwent palliative surgery only, who had prior chemotherapy, radiotherapy or were diagnosed with other primary malignancies were excluded. In addition, 30 GC patients with PM underwent palliative surgery between January 2014 and December 2018 were also enrolled in our study. Among these patients, 15 had previously undergone neoadjuvant intraperitoneal and systemic chemotherapy (NIPS), 6 had undergone other types of neoadjuvant therapy and 9 had not received any neoadjuvant treatment. The samples of these 30 patients had multiple immunofluorescence staining (EGR1, TGF-β1) and were analyzed for positive cell density (PCD). All samples were paraffin-blocked from Department of Pathology, Ruijin Hospital, Shanghai Jiao Tong University School of Medicine.

### Immunofluorescence staining

After paraffin sections were dewaxed, they were successively sealed with hydrogen peroxide and serum. The primary antibody was incubated overnight at 4 ℃, and specific protein and nuclear staining were performed with fluorescent secondary antibody and DAPI. Images were collected. PCD was defined as the number of positive cells/tissue areas in the test regions.

### Immunohistochemistry staining (IHC)

Formalin-fixed paraffin embedded (FFPE) was performed for mice peritoneal tumor tissue and then 5 μm-thick slices were incubated with the primary antibodies at 4℃ overnight. The next day, slices were incubated with HRP-labeled secondary antibodies at 37°C for 1 h, and finally all slices were visualized with diaminobenzidine. The staining intensity of each sample was scored according to the color intensity (0, no positive staining; 1, weak positive staining; 2, moderate positive staining; 3, strong positive staining) and the range of positive staining cells (1, 0%-25%; 2, 26-50%; 3, 51-75%; 4, 76-100%). Multiplied the two scores to obtain the final IHC score. At least three fields were randomly selected for each specimen.

### Cell culture and materials

The human derived peritoneal mesothelial cells (HMrSV5), NCI-N87 and SGC-7901 cell lines were purchased from the Chinese Academy of Sciences. All experimental cells were excluded mycoplasma contamination. They were cultured in Dulbecco's Modified Eagle's Medium (DMEM) supplemented with 10% fetal bovine serum (FBS), 100 U/mL penicillin and streptomycin. The incubator was maintained at 37 °C under 5% CO_2_.

### CRISPR-Cas9 and virus transfection

According to the manufacturer's instructions, lentiviruses were used to infect NCI-N87 and SGC-7901 cells to construct CD44s knockdown cells. Lentiviruses were constructed with specific primers (forward: 5'-GATCCGCGCAGATCGATTTGA-3', reverse: 5'-AATTAAAAAAGCGCAGATCGAT-3') from Genomeditech (Shanghai, China). Lentiviruses containing pcDNA3-EGR1 or cas9/sgRNA-EGR1 were separately transfected in HMrSV5. A stable EGR1 overexpressing cell line (HMrSV5-EGR1) as well as a cell line with suppressed endogenous EGR1 (HMrSV5 sgRNA EGR1) were established. Small guide RNAs (sgRNAs) were designed as follows: sgRNA#1: GGACAACTACCCTAAGCTGG, sgRNA#2: TTAGGGTAGTTGTCCATGGT, sgRNA#3: CTGCAGATCTCTGACCCGTT.

### Co-immunoprecipitation (Co-IP) and Western blot

The Thermo Scientific™ Pierce™ Co-Immunoprecipitation (Co-IP) Kit (Cat. 26149) was used to study protein:protein interactions based on covalently coupling antibodies onto an amine-reactive resin. This kit uses an antibody to immunoprecipitate the antigen (bait protein) and coimmunoprecipitate interacting proteins (prey proteins).

Western blot assays were performed as previously described 4. Briefly, protein samples were separated by 12.5% SDS-PAGE gel and were transferred to PVDF membrane using wet transfer method. They were blocked with 5% BSA and incubated with the corresponding primary antibodies overnight at 4 °C. This was followed by species-specific secondary antibody for 1 h at room temperature. Infrared imaging system (Tanon Life & Science Co.) and ECL substrate solution (Beyotime Biotechnology) for luminescence test was used. Antibodies were listed in Supplementary Table 1.

### ELISA assay

The TGF-β1 concentrations of HMrSV5 supernatants co-cultured with different EGR1 expression levels in GC cells were tested. According to the instructions, the conventional culture medium of adherent cells was replaced with serum-free culture medium and continued to culture for 48 h. Afterwards, the cell supernatants were collected for detection. The kit used a sandwich enzyme immunoassay that was pre-coated with TGF-β1 primary antibody. The absorbance (optical density, OD) value was measured at 450 nm.

### RNA-seq

We used NucleoSpin RNA Plus (Macherey-Nagel) to separate total RNA and evaluated its purity and integrity following the manufacturer's instructions. RNA was randomly fragmented by using divalent cations. Afterwards, these fragments were reverse transcribed into cDNA for terminal repair. The obtained cDNA was connected to the index connector, and the product was separated and purified. Finally, cDNA was matched through PCR amplification and cDNA library quality inspection using Illumina platform for complete computer sequencing.

### Quantitative real-time PCR analysis (qRT-PCR)

Using GAPDH as an internal reference, CT values of target genes in different cell lines were measured through the QuanStudio 6 Flex system. Total RNA of cells was extracted by TRIzol and then reverse-transcribed into 20 µL cDNA system. Mixing cDNA, primers, SYBR Green PCR MIX (Applied Biosystems) and DEPC water together according to the instructions. Finally, the relative expression of target genes in cells was calculated. All reactions were performed in triplicate. QRT-PCR primer sequences are listed in Supplementary Table 2.

### Transwell assay

In the co-culture system, up/down-regulated EGR1-HMrSV5 and their respective control HMrSV5 cells were placed on the 24-well plate (1.25 × 10^4^/well), the cells were conventionally cultured until attachment, and then the medium was replaced with serum-free medium. Afterwards, the upper compartment GC cells (5 × 10^4^/well) were planked. For invasion assay, 100 μL Matrix per well in transwell chamber (8 μm) was placed and solidificated at 37 ℃. The NCI-N87 and SGC-7901 cells were collected with the concentration at 5 × 10^4^/mL in 200 μL serum free medium. After 24 h of conventional culture, the chamber was fixed in formalin for 15 min and stained with crystal violet for 15 min. The tumor cells on the back of the chamber membrane were observed and counted under the microscope to evaluate the invasiveness of GC cells. For migration assay, no matrix was added to the transwell chamber.

### Spheroid formation assay

DMEM culture medium was replaced by stem cell culture medium (Promo Cell, Cat. C-28070). An amount of 500 μL stem cell culture medium was added into each well. A number of 2000 cells for NCI-N87 and SGC-7901 were placed into the ultra-low adhesion 24-well plate, followed by supplementation with 500 μL stem cell culture medium every 3 days. After 9 days of culture, each group of GC cells in the 24-well plate was transferred into a 15 mL centrifuge tube. PBS solution was washed and then digested with trypsin. A number of 2000 cells were counted and added to a new ultra-low adhesion 24-well plate. An amount of 1 mL stem cell culture medium was added per well. Supplementation with 500 μL stem cell culture medium was continued every 3 days to the 9th day. The tumor spheres in each group were observed under the microscope. In the co-culture system, when GC cells were plated for the second time, a number of 2000 HMrSV5 cells with EGR1 up/down-regulation or their control HMrSV5 cells were added to the upper compartment, respectively.

### Chromatin immunoprecipitation (ChIP) assay

ChIP assay was performed according to the protocol of Chromatin Immunoprecipitation Kit (Millipore, Cat.17-371). NCI-N87 and SGC-7901 cells were cross-linked with 1% formaldehyde. Subsequently, cell lysates were applied with ultrasonic treatment to produce 200-1000 bp chromatin fragments. These fragments were combined with ChIP specific antibody for immunoprecipitation. Specific primers were designed for the target fragments of ChIP DNA, Input DNA and control DNA, and qRT-PCR was performed. TGF-β1 primer sequences are listed in Supplementary Table 2.

### Dual-luciferase reporter assay

TGF-β1 promoter binding sites for EGR1 were predicted using Ensembl (http://grch37.ensembl.org/index.html) and JASPAR (http://jaspar.genereg.net). The binding sites for EGR1 in the TGF-β1 promoter is located at -146 to -125 bp and -98 to -57 bp, namely binging site 1 and 2. The wild-type TGF-β1 promoter (WT, -1900 to +100 bp), EGR1 binding site 1 mutated TGF-β1 promoter (MT1, -1900 to +100 bp), EGR1 binding site 2 mutated TGF-β1 promoter (MT2, -1900 to +100 bp) and binding sites truncated TGF-β1 promoter (MT3, -1900 to -183 bp) were cloned into the luciferase reporter vector pGL3 basic (Genomeditech). HEK 293T cells were co-transfected with the corresponding reporter plasmid in each group. The TK reporter construct was used as the internal control. The Luciferase activity was detected using the dual-luciferase report assay system.

### Peritoneal metastatic xenograft model

BALB/C nude mice were all housed in a specific pathogen-free environment. Cells used for experiments were transfected with firefly luciferase using lentivirus and were selected for resistance with puromycin. HMrSV5 and GC cells with stable luciferase expression were injected into nude mice at a 1:4 ratio (HMrSV5: 1.25 × 10^6^; NCI-N87/SGC7901: 5 × 10^6^ per mouse). Four weeks later, D-luciferin sodium salt (Yisheng biotech Co, cat. 40901es02) was injected into the peritoneal cavity at a 150 mg/kg concentration of luciferin/body weight into nude mice for 10 min, followed by the observation of tumor *in vivo* through imager. Bioluminescence intensity (BLI) was observed and statistically analyzed. Finally, the nude mice were all sacrificed to observe the number and size of intraperitoneal tumors. In the administration experiment, for SHR-1701 treatment group, HMrSV5 and GC cells with stable luciferase expression at a 1:4 ratio (HMrSV5: 6.25 × 10^5^; NCI-N87/SGC7901: 2.5 × 10^6^ per mouse). SHR-1701 (15 mg/kg/mouse) was injected into the tail vein every three days from the 8th day to the 21st day. The control group was injected with the same volume of physiological saline on each injection day. Shanghai Medical Experimental Animal Care Commission guidelines were strictly followed in all animal experiments.

### Statistical analysis

All data are presented as mean ± standard deviation (SD). The independent t-test or one-way ANOVA was used for normally distributed variables and the Mann-Whitney U test or Welch ANOVA was used for other variables. All statistical tests were two-sided and P < 0.05 was considered statistically significant (*, P < 0.05; **, P < 0.01). Statistical analysis was performed using SPSS (version 16.0) software.

## Results

### HOXA11 up-regulates the expression of EGR1 in peritoneal mesothelial cells of PM in GC patients

**Figure 1:**
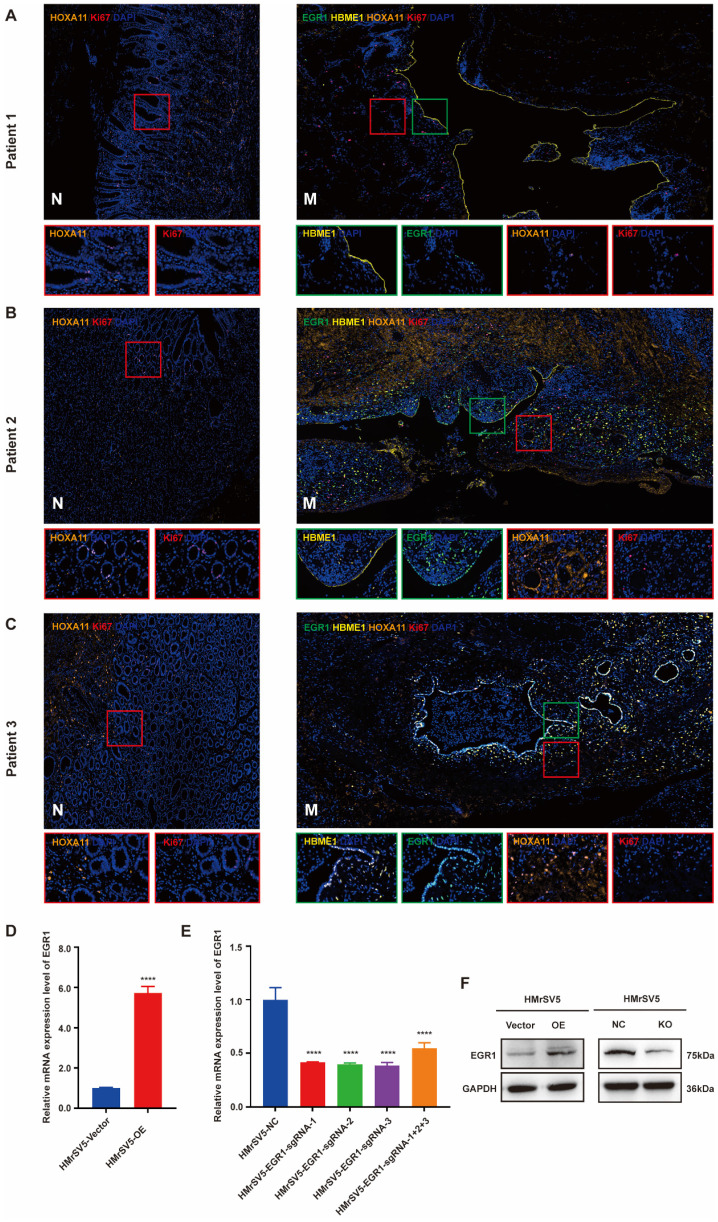
** HOXA11 up-regulates the expression of EGR1 in peritoneal mesothelial cells of PM in GC patients.** A-C. Multiple immunofluorescences staining of the PM and the matched paracancerous tissue in three patients with GC confirmed by pathology (N: paracancerous; M: peritoneal metastasis) (n=3). D. The expression level of EGR1 was determined by qRT-PCR in HMrSV5 transfected with overexpressing lentivirus and its control. E. The expression level of EGR1 was determined by qRT-PCR in HMrSV5 transfected with CRISPR-Cas9 and its control. F. Western blot analyses of the levels of EGR1 in EGR1-knockout and EGR1-overexpressing HMrSV5 cells. ****, P < 0.0001.

We collected the PM and matched paracancerous tissues of 8 patients with GC from Ruijin Hospital, Shanghai Jiao Tong University School of Medicine. All patients were diagnosed as gastric adenocarcinoma by pathology. After signing the informed consent form, the peritoneal metastatic tumor tissues were collected and fixed in formalin, then were embedded in paraffin, and prepared for slides. The expression of EGR1 in the metastatic mesothelial cells was detected by multiple immunofluorescences The correlation between EGR1 and HOXA11 in the cancerous components of the metastatic tumor was analyzed (mesothelial cells were positioned by HBME1). The results demonstrated a correlation between low expression of HOXA11 in metastatic lesions and low expression of EGR1 in mesothelial cells (Fig. 1A). In Fig. 1B and 1C, compared with non-cancerous tissues, the expression of HOXA11 in GC cells in metastasis was visibly up-regulated, which was accompanied by a high expression of EGR1 in peritoneal mesothelial cells. This is consistent with the previous results from our group. Multiple immunofluorescences of total eight patients were presented in Supplementary Fig. 1A. We also sampled 10 regions per tissue and calculated the mean fluorescence intensity (immunofluorescence intensity/area) to conduct correlation analysis between HOXA11 and EGR1. The spearman correlation analysis showed a positive correlation result (Supplementary Fig. 1B). Thus, these findings prompted that the overexpression of HOXA11 in GC cells may induce EGR1 upregulation in HPMC. HMrSV5 is a commercial humanized peritoneal mesothelial cell. To verify the role of EGR1 in GC PM, we first used lentivirus transfection and CRISPR-Cas9 technology to construct stable EGR1 overexpression and knock-out peritoneal mesothelial cell models *in vitro*. The expression level of EGR1 was examined by qRT-PCR and Western blot assays (Fig. 1D-F).

### EGR1 in peritoneal mesothelial cells promotes the stemness, migration and invasion of GC cells *in vitro* and *in vivo*

**Figure 2:**
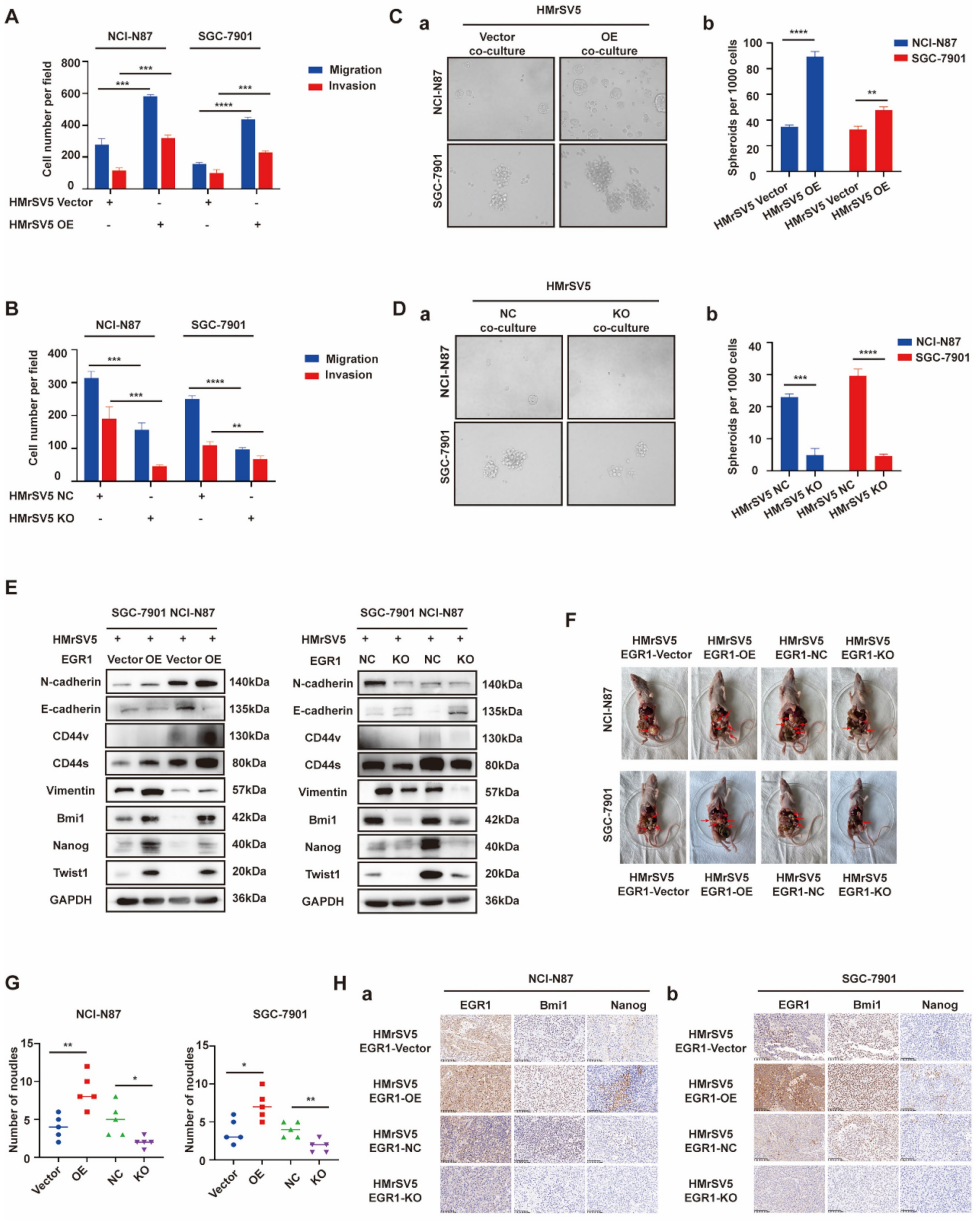
** EGR1 in peritoneal mesothelial cells promotes the stemness, migration and invasion of GC cells *in vitro* and *in vivo*.** A-B. Cell migration and invasion were analyzed in NCI-N87 and SGC-7901 co-cultured with EGR1-overexpressing HMrSV5 cells (A) and EGR1-knockout HMrSV5 cells (B) (n=3). C-D. Spheroid-forming ability was analyzed in NCI-N87 and SGC-7901 co-cultured with EGR1-overexpressing HMrSV5 cells (Ca-b) and with EGR1-knockout HMrSV5 cells (Da-b) (n=3). E. Western blot analyses of the levels of stemness- and EMT-related biomarkers in NCI-N87 and SGC-7901 co-cultured with EGR1-overexpressing and EGR1-knockout HMrSV5 cells. F. Representative mice imaging of peritoneal metastatic model formed by NCI-N87 or SGC-7901 co-injected with different EGR1-expressing HMrSV5 cells (n=5). G. Number of peritoneal metastatic nodules *in vivo* (n=5). Ha-b. The expression of EGR1 and stemness biomarkers Bmi1 and Nanog of xenografts from peritoneal metastatic nodules formed by NCI-N87 or SGC-7901 co-injected with different EGR1-expressing HMrSV5 cells. **, P < 0.01; ***, P < 0.001; ****, P < 0.0001.

To verify the role of EGR1 in GC peritoneal microenvironment remodeling, HMrSV5 cells with different levels of EGR1 expression were employed to co-culture with GC cells (NCI-N87/SGC-7901) for loss- or gain-of-function studies. The results indicated that GC cells exhibited enhanced migration and invasion in the co-culture system with EGR1 up-regulated HMrSV5, while the migration and invasion of GC cells decreased when co-cultured with EGR1 down-regulated HMrSV5 (Fig. 2A-B; Supplementary Fig. 2A-B). Additionally, we found that GC cells co-culturing with EGR1 up-regulated HMrSV5 resulted in a notable increase in their spheroid-forming ability. The down-regulation of EGR1 in HMrSV5 inhibited the spheroid-forming ability of GC cells in co-culture system (Fig. 2C-D). We also evaluated the protein expression of epithelial-mesenchymal transformation (EMT) (N-cadherin, E-cadherin, Vimentin, Twist1) and cell stemness-related biomarkers (Bmi1, Nanog, CD44) in GC cells that were co-cultured with EGR1-overexpressing and EGR1-knockout HMrSV5 cells. The findings indicated that EGR1 expression altering in mesothelial cells had an impact on the expression of GC cell stemness and EMT biomarkers (Fig. 2E).

Following our investigation into the impact of EGR1 on GC cell phenotypes *in vitro*, we proceeded to study its effects on GC peritoneal tumorigenicity *in vivo* using mouse xenograft models. HMrSV5 cells with high/low expression level of EGR1 were injected into the abdominal cavity of nude mice with GC cells at a ratio of 1:4. Compared with the control group, tumor metastases were significantly increased in models which GC cells were simultaneously inoculated with EGR1-overexpressing mesothelial cells. Conversely, the tumor metastases were significantly reduced in EGR1-knockout mesothelial cells and GC cells inoculated group (Fig. 2F-G). In addition, our results showed that the expression of Bmi1 and Nanog, the stemness biomarkers of GC cells, were positively correlated with the expression of EGR1 in mesothelial cells (Fig. 2H). The statistical analyses of EGR1, Bmi1 and Nanog expression were evaluated by IHC score in tissues of xenografts (Supplementary Fig. 2C).

### EGR1 regulates GC cell phenotypes by directly binding to TGF-β1 promoter

**Figure 3:**
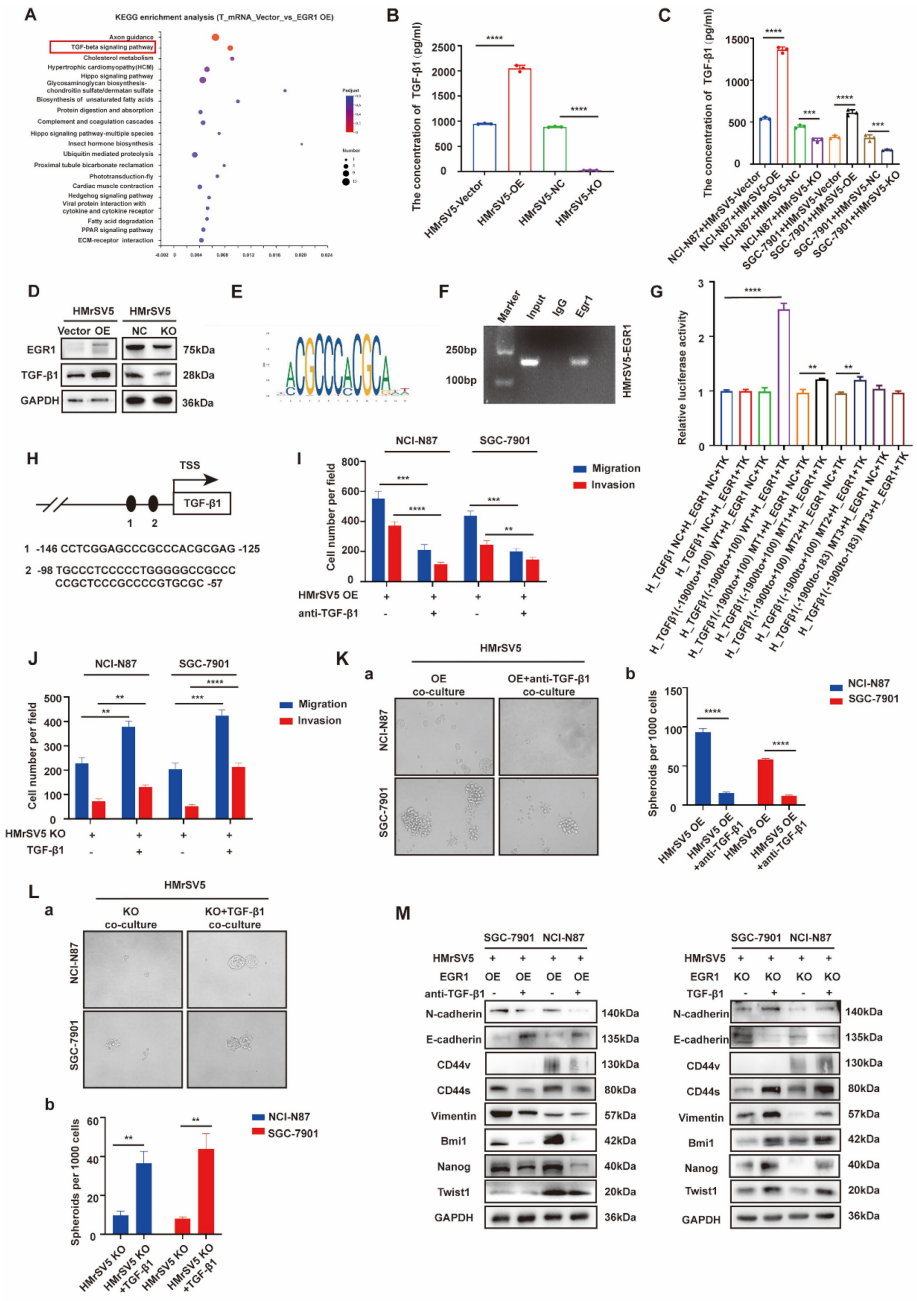
** EGR1 regulates GC cell phenotypes by directly binding to TGF-β1 promoter.** A. KEGG pathway enrichment analysis between EGR1-overexpressing HMrSV5 cells and their control group. B. Detection of TGF-β1 concentration in HMrSV5 with different EGR1 expression levels (n=3). C. Detection of TGF-β1 concentration in co-culture system with GC cells and different EGR1 expressed HMrSV5 (n=3). D. Western blot analyses of the levels of TGF-β1 in HMrSV5 with different EGR1 expression levels. E. EGR1 binding motif. F. Agarose electrophoresis for ChIP analysis of EGR1 binding to the TGF-β1 promoter. G. HMrSV5 were transfected with wild type or mutation type TGF-β1 luciferase reporter vector. The relative luciferase activities were determined by the Dual-Luciferase Reporter Assay (n=3). H. JASPAR analysis showed two potential EGR1-binding sites of the TGF-β1 promoter. I-J. Cell migration and invasion were analyzed in NCI-N87 and SGC-7901 treated with TGF-β1 neutralizing antibody in EGR1-overexpressing HMrSV5 cells co-culture system (I) and with recombinant TGF-β1 in EGR1-knockout HMrSV5 cells co-culture system (J) (n=3). K-L. Cell spheroid formation ability was analyzed in NCI-N87 and SGC-7901 treated with TGF-β1 neutralizing antibody in EGR1-overexpressing HMrSV5 cells co-culture system (Ka-b) and with recombinant TGF-β1 in EGR1-knockout HMrSV5 cells co-culture system (La-b) (n=3). M. Western blot analyses of the levels of stemness- and EMT-related biomarkers in NCI-N87 and SGC-7901 treated with TGF-β1 neutralizing antibody or recombinant TGF-β1 in co-culture system with EGR1-overexpressing and EGR1-knockout HMrSV5 cells. **, P < 0.01; ***, P < 0.001; ****, P < 0.0001.

To explore the mechanism of EGR1 acts in HMrSV5, we performed mRNA transcriptome sequencing between EGR1 overexpressed HMrSV5 cells and their control group. Kyoto Encyclopedia of Genes and Genomes (KEGG) pathway enrichment analysis showed that EGR1 could regulate TGF-β signaling pathway in GC cells (Fig. 3A). Recent studies have also shown that EGR1 transcriptional-modulated TGF-β1 drives the occurrence of fibrosis or various tumors. Therefore, we assessed the concentration of TGF-β1 in HMrSV5 supernatant with different EGR1 expression levels. The results showed that overexpression of EGR1 increased TGF-β1 secretion in mesothelial cells. Conversely, the absence of EGR1 resulted in decreased TGF-β1 secretion in mesothelial cells (Fig. 3B). These findings were also valid in the co-culture system of HMrSV5 and GC cells (Fig. 3C). Additionally, Western blot analysis was performed to measure the TGF-β1 expression in HMrSV5 cells with different EGR1 levels. The results were consistent with the ELISA assay, indicating a positive correlation between EGR1 and TGF-β1 protein expression (Fig. 3D).

Based on JASPAR, there are two potential EGR1 binding sites on the promoter of TGF-β1 (Fig. 3E). To evaluate the effect of EGR1 on TGF-β1 in HMrSV5 cells, ChIP assays were performed and the findings demonstrated that EGR1 directly binds to the TGF-β1 promoter in HMrSV5 cells (Fig. 3F). The ChIP primer included both binding sites simultaneously due to their close proximity. Dual-luciferase reporter assays were also performed to identify regulatory elements that control TGF-β1 transcription. The results showed that overexpression of EGR1 increased TGF-β1 promoter activity through both two EGR1 binding sites (Fig. 3G-H).

To clarify whether the effect of EGR1 on the migration, invasion and stemness of GC cells depends on the secretion of TGF-β1 in the co-culture system *in vitro*, we conducted a pro- and con- verification by artificially interfering with the TGF-β1. In transwell assays, first, TGF-β1 neutralizing antibody (20 µg/mL) was used to act on GC cells co-cultured with EGR1-highly expressed HMrSV5. It was found that the originally enhanced migration and invasion of GC cells were weakened. Conversely, when human recombinant TGF-β1 (20 ng/mL) was used to act on GC cells co-cultured with EGR1-knockout HMrSV5, the ability of migration and invasion was enhanced compared with the control group (Fig. 3I-J; Supplementary Fig. 3A-B). In the spheroid formation assays, the results obtained by adding TGF-β1 neutralizing antibody (20 µg/mL) and human recombinant TGF-β1 (20 ng/mL) were consistent with the above results, where the spheroid formation ability of GC cells was found to be significantly weakened when TGF-β1 was suppressed (Fig. 3K) and significantly strengthened when exogenous TGF-β1 was applied (Fig. 3L). Additionally, we evaluated the presence of EMT and cell stemness-related biomarkers in GC cells treated with TGF-β1 neutralizing antibody (20 µg/mL) and human recombinant TGF-β1 (20 ng/mL) in the co-culture system. The results suggested that the alteration of TGF-β1 due to EGR1 overexpression or down-regulation in mesothelial cells was the primary cause for the modification of GC cell stemness- and EMT-related biomarkers' expression (Fig. 3M).

### EGR1 regulates stemness and EMT phenotypes of GC cells by impacting with CD44s

**Figure 4:**
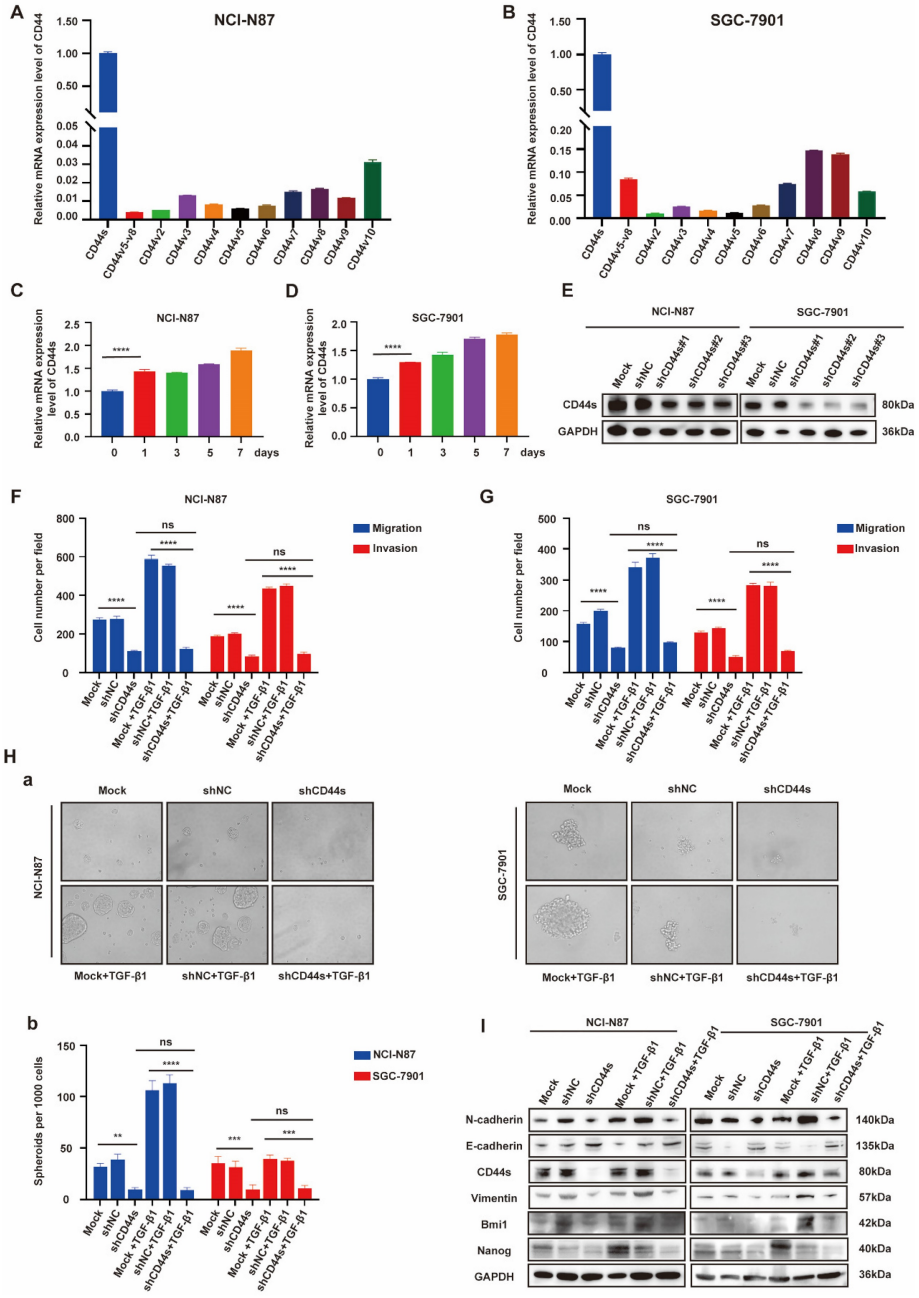
**EGR1 regulates stemness and EMT phenotypes of GC cells by impacting with CD44s.** A-B. The expression levels of different CD44 splicing variants were determined by qRT-PCR in NCI-N87 and SGC-7901. C-D. The one-week expression levels of CD44s were determined by qRT-PCR in NCI-N87 and SGC-7901 co-cultured with EGR1-overexpressing HMrSV5 cells. E. Western blot analyses of the levels of CD44s in CD44s knockdown NCI-N87 and SGC-7901 cells. F-G. Cell migration and invasion were analyzed in CD44s-knockdown NCI-N87 (F) and SGC-7901 (G) cells and their respective control groups co-cultured in the presence or absence of TGF-β1 (n=3). Ha-b. Cell spheroid formation ability was analyzed in CD44s knockdown NCI-N87 and SGC-7901 cells and their respective control groups co-cultured in the presence or absence of TGF-β1 (n=3). I. Western blot analyses of the levels of stemness- and EMT-related biomarkers in GC cells (NCI-N87 and SGC-7901) transfected with CD44s shRNA and negative control shRNA co-cultured in the presence or absence of TGF-β1. **, P < 0.01; ***, P < 0.001; ****, P < 0.0001.

Cancer stem cells (CSCs) have been characterized in a variety of tumors, including GC, colorectal cancer, and pancreatic cancer. These cells, believed to fuel tumor growth and promote metastasis, are important treatment targets 16-19. EMT, representing a phenotypic plasticity, plays an important role in the process of tumor metastasis 20. Cancer stemness and EMT are complex manifestations of cellular plasticity driven by multiple cross signal pathways. Cell adhesion molecule CD44 is a widely recognized stem cell biomarker and has been proved to be a functional intersection between signal networks that regulate stemness and epithelial mesenchymal plasticity in a variety of solid tumors 21. Thus, in the next step, we evaluated whether EGR1 promotes stemness and EMT phenotypes of GC cells through CD44 regulation. It is known that CD44 is diversely expressed as multiple splicing variants. Therefore, we first detected the basic mRNA expression level of each variant in NCI-N87 and SGC-7901 cells by qRT-PCR. The results showed that the standard CD44 (CD44s) transcriptional level was significantly higher than other variants (Fig. 4A-B). At the meantime, we co-cultured EGR1-overexpressing HMrSV5 with NCI-N87 and SGC-7901 for one week and measured the expression levels of CD44s in GC cells every two days. The results indicated an upward trend of CD44s in GC cells throughout the experiment in this co-culture system (Fig. 4C-D). Therefore, we chose CD44s for exploring the downstream mechanism, and constructed NCI-N87 and SGC-7901 cell lines with CD44s knockdown via lentivirus transfection (Fig. 4E). To confirm CD44s' vital role in the stemness and EMT phenotypes of EGR1-regulated GC cells, we performed transwell and spheroid formation assays after knocking down CD44s. The transwell results revealed that CD44s knocking down noticeably impaired the migration and invasion capabilities of GC cells in comparison to the control group. At the presence of 20 ng/mL humanized TGF-β1, GC cells displayed a noticeable enhancement in migration and invasion in the control group, which was not observed in the CD44s-knockdown group (Fig. 4F-G; Supplementary Fig. 4A-B). In spheroid formation assays, the trend of cell stemness was consistent with that observed in the transwell assays. The spheroid formation ability of GC cells weakened when the expression of CD44s was reduced, and co-culture with TGF-β1 (20 ng/mL) did not restore cell stemness (Fig. 4H). In addition, we evaluated the expression of EMT- and cell stemness-related biomarkers in GC cells cultured alone or stimulated with TGF-β1 (20 ng/mL), with or without CD44s knockdown. The results indicated that CD44s was the main downstream of EGR1-TGF-β1 induced GC cell plasticity (Fig. 4I).

### EGR1 regulates GC cell phenotypes via the TGF-β1/CD44s/STAT3 pathway

**Figure 5:**
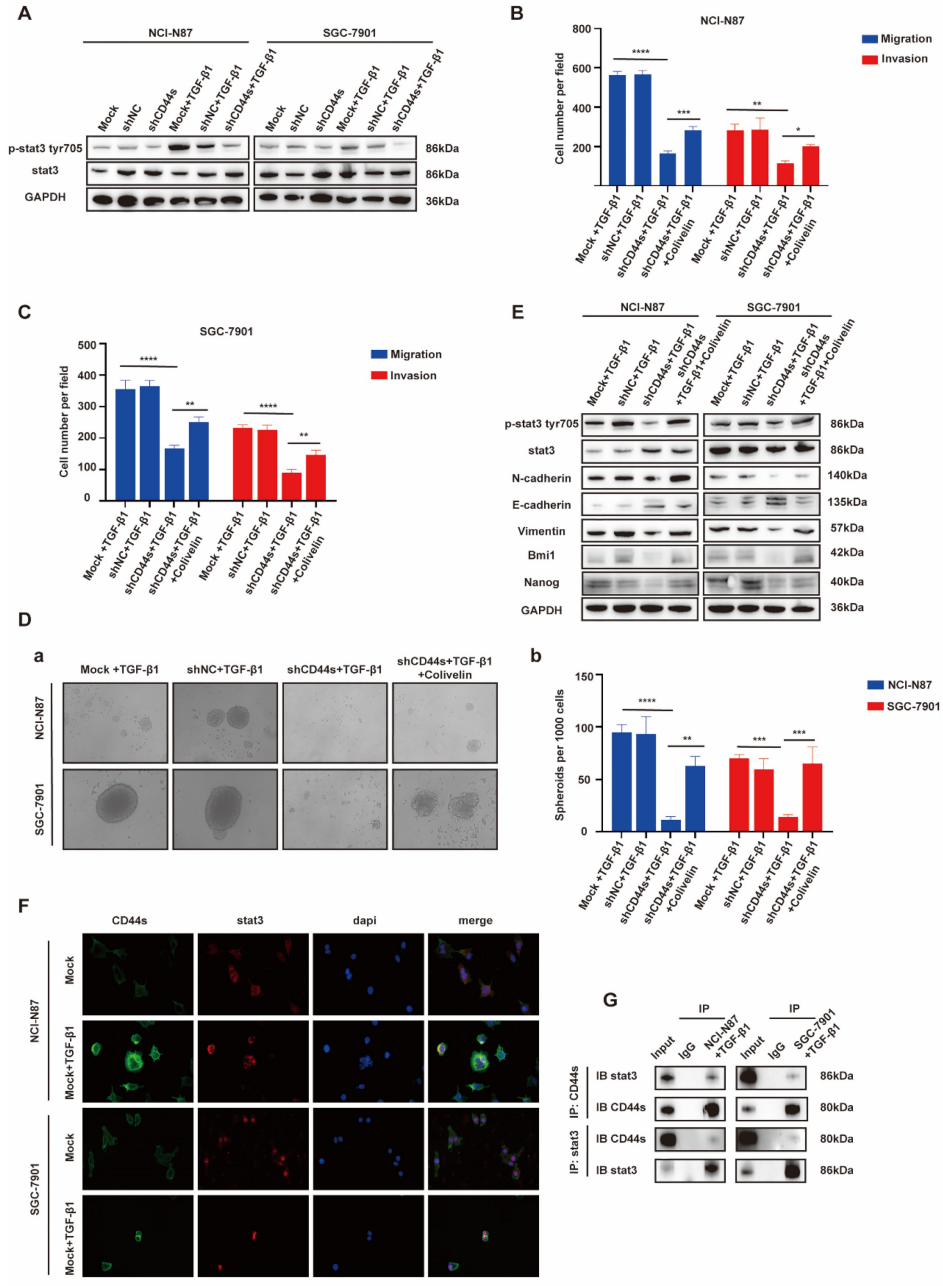
**EGR1 regulates GC cell phenotypes via the TGF-β1/CD44s/STAT3 pathway.** A. Western blot analyses of the levels of STAT3 and p-STAT3 in CD44s knockdown NCI-N87 and SGC-7901 cells and their respective control groups co-cultured with TGF-β1. B-C. Cell migration and invasion were analyzed in CD44s knockdown NCI-N87 (B) and SGC-7901 (C) cells and their respective control groups treated in the presence or absence of Colivelin in TGF-β1 co-culture system (n=3). Da-b. Cell spheroid formation ability was analyzed in CD44s knockdown NCI-N87 and SGC-7901 cells and their respective control groups treated in the presence or absence of Colivelin in TGF-β1 co-culture system (n=3). E. Western blot analyses of the levels of STAT3, p-STAT3, stemness- and EMT-related biomarkers in GC cells (NCI-N87 and SGC-7901) transfected with CD44s shRNA and negative control shRNA treated in the presence or absence of Colivelin in TGF-β1 co-culture system. F. Immunofluorescence assay evaluating the cell internalization of CD44s and its interaction with STAT3 in NCI-N87 and SGC-7901 cells in the presence or absence of TGF-β1 stimulation. G. Endogenous CD44s or STAT3 was immunoprecipitated with anti-STAT3 or anti-CD44s antibody, and immunocomplexes were analyzed with Western blot with anti-STAT3 and anti-CD44s antibodies. *, P < 0.05; **, P < 0.01; ***, P < 0.001; ****, P < 0.0001.

STAT3 is a pleiotropic transcription factor, which participates in the expression of a variety of stemness-related target genes 22, 23. Activated STAT3 is a key dedicator to tumor survival, proliferation, invasion and metastasis. In the past decades, several studies have shown that CD44 and STAT3 cooperate with each other in cancer promotion 24. Therefore, we hypothesized that CD44s could maintain the stemness- and EMT-related phenotypes of GC cells by regulating STAT3 activation in our model. To begin with, we determined whether the change of CD44s expression affects STAT3 phosphorylation in GC cells by Western blot. We found that CD44s knockdown reduced the phosphorylation level of STAT3 in GC cells. However, TGF-β1 (20 ng/mL) could not rescue the decrease of STAT3 phosphorylation level caused by CD44s knockdown (Fig. 5A). To validate the involvement of STAT3 in the stemness, migration and invasion phenotypes of CD44s-promoted GC cells, a STAT3 activator (Colivelin) was administered to CD44s-knockdown GC cells. In transwell assays, compared with the control group, CD44s-knockdown GC cells stimulated with TGF-β1 (20 ng/mL) showed a significantly reduced migration and invasion ability. Nevertheless, the addition of 50 µg/mL Colivelin could reverse the EMT phenotype induced by CD44s knockdown (Fig. 5B-C; Supplementary Fig. 5A-B). The changes in cell stemness observed in the spheroid formation assays matched the results of the transwell experiment. These findings implied that the addition of Colivelin (50 µg/mL) could reverse the reduction in stemness caused by CD44s knockdown (Fig. 5D). For additional verification, we evaluated the expression levels of stemness- and EMT-related biomarkers in GC cells treated with Colivelin (50 µg/mL). The results showed that Colivelin caused an increase of STAT3 phosphorylation, along with the up-regulation of stemness and EMT biomarkers (Fig. 5E).

The above results indicated that CD44s knockdown could down-regulate STAT3 phosphorylation level in GC cells, and the inhibition of STAT3 activation directly repressed the stemness, migration and invasion of GC cells. Furthermore, the direct intracellular interaction between CD44 and STAT3 has been reported in cancers 25, 26. Therefore, we used Co-IP and immunofluorescence assays to explore whether there was an interaction between CD44s and STAT3 in this model. The results showed an increase in CD44s expression, with internalization and co-localization with STAT3 in NCI-N87 and SGC-7901 cells activated by TGF-β1 (20 ng/mL) (Fig. 5F). By immunofluorescence, we also found that ligation of CD44s promoted nuclear colocalization of CD44s and p-STAT3 in NCI-N87 and SGC-7901 cells (Supplementary Fig. 5C). Co-IP assays provided further evidence that CD44s combined with STAT3 to promote STAT3 nuclear translocation and phosphorylation in a high TGF-β1 environment (Fig. 5G).

### EGR1 in peritoneal mesothelial cells up-regulates the expression of CD44 in GC tissues

**Figure 6:**
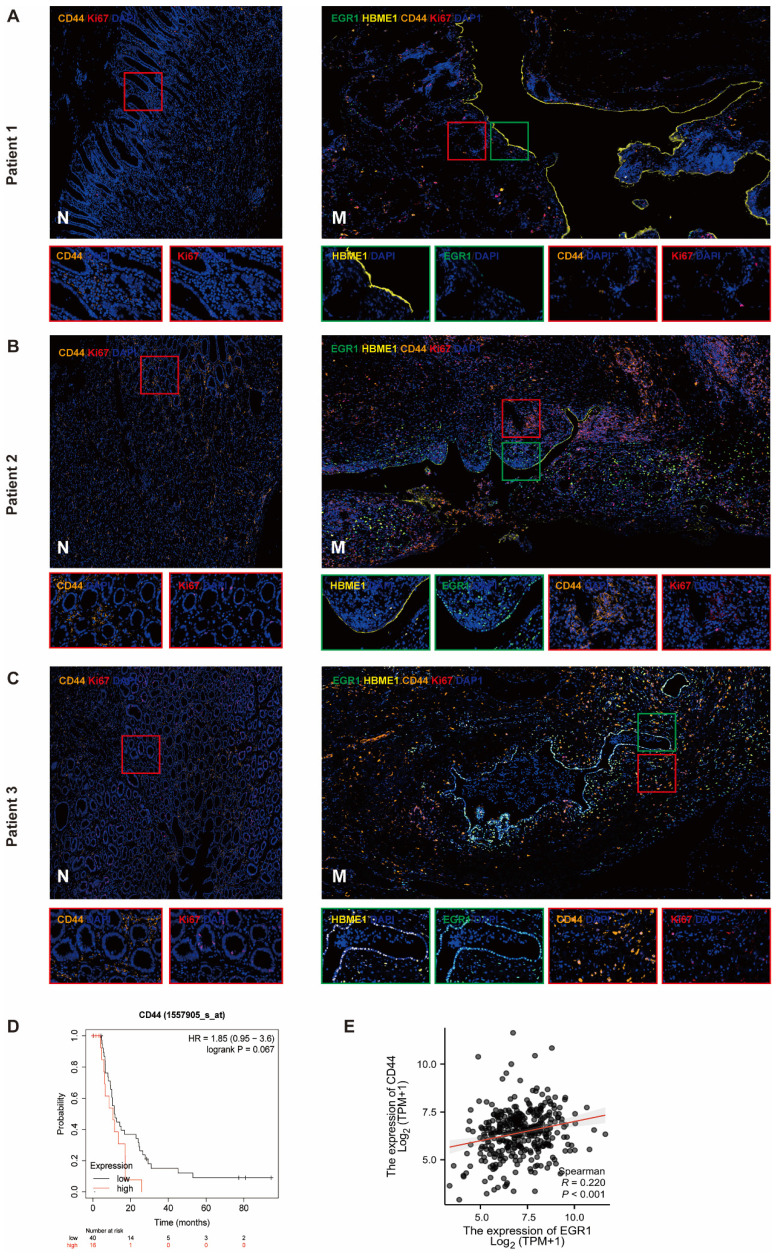
**EGR1 in peritoneal mesothelial cells up-regulates the expression of CD44 in GC tissues.** A-C. Multiple immunofluorescences staining of the PM and the matched paracancerous tissue in three selected patients with GC confirmed by pathology (N: paracancerous; M: peritoneal metastasis) (n=3). D. Kaplan-Meier survival analysis between the low and high expression groups of CD44 in M1 stage GC tissues. E. Correlation analysis between EGR1 and CD44 in human GC tissues based on TCGA database.

We detected the expression of CD44 in tumor cells of the metastases by multiple immunofluorescences and analyzed the correlation between EGR1 and CD44 in the tumor components of the metastatic tumor (mesothelial cells were positioned by HBME1). The results showed that the CD44 levels in metastatic tissues were higher than that in non-cancer tissues. In our observation, we found a positive correlation between increased EGR1 expression in metastatic lesions and increased CD44 expression in peritoneal mesothelial cells in GC tissues. This is consistent with our previous findings. These findings suggested that the overexpression of EGR1 in peritoneal mesothelial cells induced the up-regulation of CD44 in GC cells (Fig. 6A-C; Supplementary Fig. 6A-B). Based on seven independent GC datasets (GSE22377, GSE14210, GSE51105, GSE62254, GSE38749, GSE15459, GSE29272), 56 GC patients with M1 stage were collected in kmplot website. Kaplan Meier survival analysis was used to evaluate the effect of different CD44 expression levels on the survival of patients with advanced GC. The result exhibited marginally significant that higher expression of CD44 was associated with poorer prognosis in M1 GC patients (Fig. 6D). Moreover, based on TCGA database, EGR1 and CD44 also showed a positive correlation in GC samples (Fig. 6E).

### EGR1/TGF-β1 in mesothelial cells promotes PM of GC *in vivo* through CD44s/STAT3 activation in GC cells

**Figure 7:**
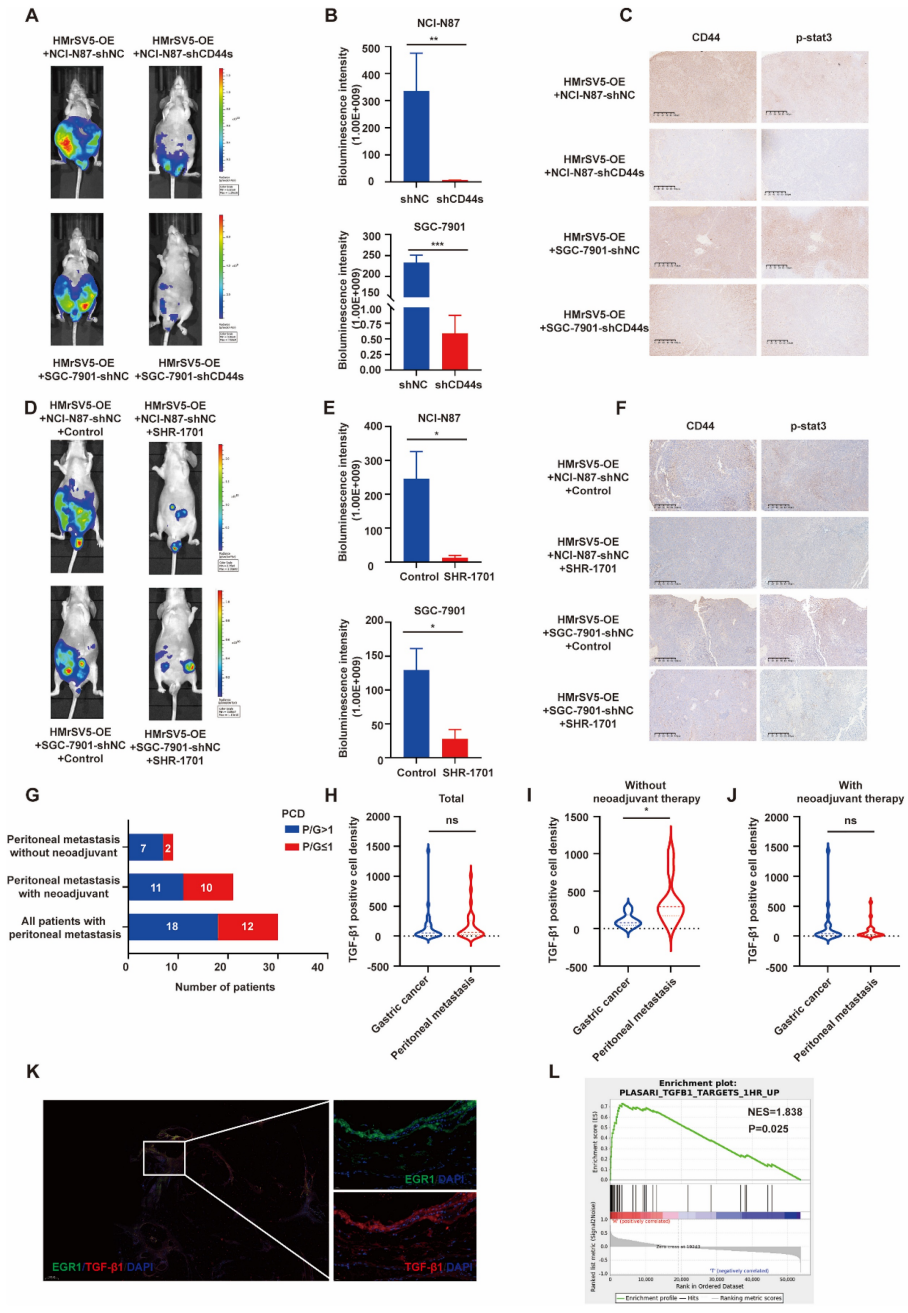
**Elevated TGF-β1 plays a significant role in PM of GC *in vivo*.** A. Representative vivo imaging of peritoneal metastatic model formed by different CD44s-level NCI-N87 or SGC-7901 co-injected with EGR1-overexpressing HMrSV5 cells (n=5). B. Semi-quantitative bioluminescence intensity of mice peritoneal metastatic models formed by different CD44s-level NCI-N87 or SGC-7901 co-injected with EGR1-overexpressing HMrSV5 cells (n=5). C. The expression of CD44 and p-STAT3 of xenografts. D. Representative *in vivo* imaging of peritoneal metastatic model formed by NCI-N87/SGC-7901 and EGR1-overexpressing HMrSV5 cells with and without SHR-1701 treatment (n=5). E. Semi-quantitative bioluminescence intensity of mice peritoneal metastatic model formed by NCI-N87/SGC-7901 and EGR1-overexpressing HMrSV5 cells with and without SHR-1701 treatment (n=5). F. The expression of CD44 and p-STAT3 of xenografts. G. Distribution of patient number with different PCD ratios of GC patients with PM (n=30). H. Comparison of TGF-β1 PCD values between primary and PM in 30 GC patients (n=30). I. Comparison of TGF-β1 PCD values between primary and PM in 9 GC patients without neoadjuvant therapy (n=9). J. Comparison of TGF-β1 PCD values between primary and PM in 21 GC patients with neoadjuvant therapy (n=21). K. Representative immunofluorescence image of GC PM mesothelial cells EGR1/TGF-β1 (n=30). L. GSEA enrichment diagram of PM vs primary lesion in 12 GC patients who had not received neoadjuvant therapy (n=12). *, P < 0.05; **, P < 0.01; ***, P < 0.001.

Following our investigation on the impact of EGR1 on CD44s/STAT3 in GC cells *in vitro*, we examined the influence of EGR1 in mesothelial cells on GC peritoneal metastasis using xenograft models. In line with our *in vitro* data, the PM of mice in the control group of EGR1-overexpressing HMrSV5 combined with NCI-N87 or SGC-7901 was significantly more severe than that of mice modeled with the EGR1-overexpressing HMrSV5 combined with NCI-N87 or SGC-7901 CD44s-knockdown cells (Fig. 7A-B). Moreover, the IHC staining indicated a significant decrease in STAT3 phosphorylation in NCI-N87 or SGC-7901 cells with CD44s knockdown (Fig. 7C). The statistical analyses of CD44 and p-STAT3 expression were evaluated by IHC score in xenograft tissues (Supplementary Fig. 7A). SHR-1701 is the first domestic anti-PD-L1/TGF-β RII bifunctional fusion protein entered clinical trials in China 27, 28. As an essential component of TGF-β1 signal transduction, TGF-β RII plays a vital role in activating the downstream pathway and altering the trend of cancer development caused by TGF-β1. We tested the possibility of using SHR-1701 to block the PM of GC by reversing the tumor microenvironment of high TGF-β1 in PM. The results demonstrated an effective control of PM in mice of the SHR-1701 group treated with EGR1-overexpressing HMrSV5 and NCI-N87 or SGC-7901 compared to the unused group (Fig. 7D-E). Moreover, the blockade of TGF-β1 inhibited the transcription of CD44s in GC cells and phosphorylation of STAT3 in the downstream (Fig. 7F). The statistical analyses of CD44 and p-STAT3 expression were evaluated by IHC score in xenograft tissues (Supplementary Fig. 7B). These results illustrated that TGF-β1 exerted a promoting effect in the occurrence of PM of GC.

### Elevated TGF-β1 is associated with PM of GC

In recent years, many studies have provided substantial evidence for TGF-β1-mediated metastatic spread of GC, indicating that TGF-β1 can create a microenvironment that is permissive to metastatic dissemination, and contribute to local invasion and colonization of distant organs 29-31. Through the results of our study, we have confirmed that mesothelial cells of GC peritoneal metastases could construct a high TGF-β1 microenvironment. Further, we studied 30 patients who underwent GC palliative operation at Ruijin Hospital from 2014 to 2018. For 30 GC samples and their matched peritoneal metastatic samples, TGF-β1 immunofluorescence and semi-quantitative analyses were performed. In 60% (18 of 30) of cases, metastases had greater TGF-β1 PCD than the primary lesions. After excluding patients who had previously received neoadjuvant therapy, 77.8% (7 of 9) of metastases had greater TGF-β1 PCD than the primary lesions (Fig. 7G). The statistical analysis of TGF-β1 PCD levels in tissues revealed that there was no difference in TGF-β1 staining level between the primary and metastatic lesions in 30 patients with GC PM (Fig. 7H). However, the TGF-β1 staining of the metastatic lesion in 9 GC patients with PM who did not receive neoadjuvant treatment was significantly higher than that of the primary lesions (Fig. 7I), while the TGF-β1 staining of the remaining 21 patients who received neoadjuvant treatment still showed no difference in PCD levels (Fig. 7J). A representative image of PM in a GC patient without neoadjuvant treatment was showed in Fig. 7K, where the results presented that peritoneal mesothelial cell simultaneously expressed high levels of EGR1 and TGF-β1. Furthermore, we collected another cohort of 12 pairs of primary GC and PM patients without new adjuvant treatment in our hospital to perform RNA-seq. The GSEA analysis revealed that the metastatic lesions had an enrichment of differential genes in the TGF-β1-related pathway, consistent with our findings (Fig. 7L).

## Discussion

Our previous research has uncovered the specific molecular mechanism of certain hub genes involved in the regulatory process during PM of GC, focusing on the role of tumor cells as seeds in seed back-feeding soil. We found that HOXA11 not only regulates the malignant phenotype of GC cells, but also enhances the adhesion of GC cells to mesothelial cells and induces MMT. Additionally, the positive feedback loop between HOXA11 and STAT3 in GC cells further exacerbates the PM caused by HOXA11 4. We also highlighted the role of tumor associated fibroblasts in promoting GC invasion and metastasis. This was achieved through the secretion of HGF and IL6, activating the IL-6R-JAK2-STAT3 pathway in GC cells. Additionally, HGF secretion stimulated PI3K/AKT and ERK1/2 pathways, leading to the formation of vascular mimicry in GC cells 32, 33. In the present study, we aimed to utilize the "soil nourish seed" perspective to confirm that up-regulating mesothelial EGR1 enhances the stemness and invasiveness of GC cells through TGF-β1, further supporting the "seed and soil" theory for PM of GC.

In this study, we confirmed that EGR1 in peritoneal mesothelial cells was up-regulated in PM of GC. Furthermore, EGR1 increased GC cell stemness, promoting PM via regulating TGF-β1 transcription and secretion. Specifically, EGR1 stimulated the expression of CD44s in GC cells via TGF-β1. Then, the membrane protein CD44s promoted the structural activation of STAT3 through the interaction with STAT3. The activation of STAT3 is involved in the expression of stemness- and EMT-related hub genes in downstream in GC cells. Multiple technical means utilized in clinical or xenograft samples supported the high TGF-β1 tumor microenvironment in PM of GC, which provided evidence to the clinical significance of our study. The Kaplan-Meier survival analysis of EGR1, TGF-β1 and STAT3 based on the M1 GC tissues through https://kmplot.com website and their correlation based on the human GC TCGA database are shown in the Supplementary Fig. 8A-F. Thus, these results serve to reinforce our findings. To our knowledge, our research is the first to propose the crosstalk of EGR1/TGF-β1 and CD44s/STAT3 communication between peritoneal mesothelial cells and tumor cells in GC.

Moreover, we observed that co-culturing EGR1-overexpressing mesothelial cells with GC cells led to a significant increase in the expression of stemness markers and EMT markers in GC cells through enhanced TGF-β1 transcription. Previous studies have shown that TGF-β1 signal pathway plays an extremely important role in EMT in various epithelial cells and transgenic mice via Smad2/3 dependent pathway 34, 35. Furthermore, several studies have underlined how TGF-β1 has a positive role in the CSC population promotion in various types of malignancies including GC 29, 36. Therefore, TGF-β1 can be viewed as an important cytokine, which plays a synergistic role in EMT and stemness sustaining of GC. Previous studies have described how TGF-β1 boosts CD44 expression in GC cells, enhancing tumor cell adhesion to peritoneum 37. Also, the recognition of CD44 as a stem cell marker of GC has been fully studied 38, where the CD44 standard subtype (CD44s) and various variant subtypes (CD44v) have been confirmed to be closely related to the malignant transformation of GC 39-41. Furthermore, it has been found that the high expression of CD44 is also closely related to GC cell motility 42. Identifying CD44s as the main form of CD44 mRNA expression in GC cells, we hypothesized CD44s could play an important role in mesothelial cell-derived TGF-β1-inducing EMT and maintaining GC stemness phenotypes, which were demonstrated to be true through our results. STAT3 is a multifunctional transcription factor, which is involved in the transcription and expression of EMT- and stemness-related biomarkers in GC and multiple other cancers 23, 43, 44. Also, some studies have shown that CD44 and STAT3 cooperate with each other in cancer promotion. For example, in colon cancer, nuclear CD44 can bind acetylated STAT3 and promote target gene expression 25; IL6/STAT3 signal transduction can participate in cell proliferation, invasion and metastasis by promoting CD44 expression in GC 45. In breast cancer and bladder cancer, CD44 knockdown inhibits cell invasion and tumorigenicity by blocking STAT3 phosphorylation. Based on the results of Co-IP experiments, the C-terminal domain of CD44 and the coil domain of STAT3 have been found to play an important role in the formation of CD44/STAT3 complex 46, 47. This suggests that STAT3 is the downstream response molecule of CD44s, making it a suitable candidate for studying cellular mechanisms.

It is known that the distant colonization of cancer cells is not only due to the proximity of the anatomical location of the primary tumor to the secondary site, but also involves the interaction between tumor cells and the local microenvironment 48. The peritoneal stroma microenvironment promotes tumor cell adhesion to the peritoneal mesothelium by providing various growth factors and chemokines to promote tumor metastasis 49. Previous studies have shown that the level of peritoneal lavage fluid TGF-β1 in GC patients is significantly correlated with PM and tumor, node, metastasis (TNM) staging of GC, and the interaction between GC cells and mesothelial cells plays a vital role in inducing GC spread 50, 51. Our study further confirmed the key contribution of TGF-β1 in PM of GC through examining multiple immunofluorescence and transcriptome sequencing of several dozens of GC patients with primary GC and matched PM. SHR-1701, as a new fusion protein which can block the extracellular domain of TGF-β1 receptor II, showed an encouraging antitumor activity in GC 27. We further demonstrated the significance of TGF-β1 in PM of GC through the first application of SHR-1701 in GC PM xenograft models, which further elevated the clinical translation significance of our study.

No doubtfully, the current study is still in its early stage when it comes to investigating the mechanism of TGF-β1 activated CD44s/STAT3 in GC cells. Also, research on SHR-1701 blocking PM in GC is limited to xenograft models, and there is still a long way to go with clinical trials.

## Conclusions

**Figure 8:**
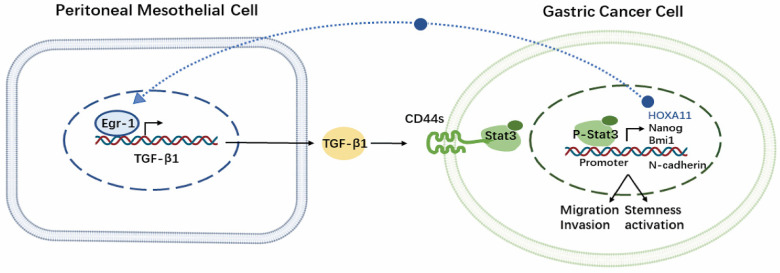
Schematic representation of the EGR1/TGF-β1 and CD44s/STAT3 crosstalk in PM of GC.

We confirmed that the crosstalk between EGR1/TGF-β1 and CD44s/STAT3 in PM of GC that was mediated by HPMCs and GC cells. Additionally, our findings demonstrated that the PM of GC was exposed to a high TGF-β1 environment, and *in vivo* experiments with SHR-1701 confirmed the inhibitory effect of TGF-β1 blockade on PM. These findings provide evidences for the essential role of "soil" nourishing "seed" theory in PM (Fig. 8), which can deepen our understanding regarding the role of microenvironment modification of PM and the overall prevention and treatment of metastasis in GC.

## Supplementary Material

Supplementary figures and tables.

## Abbreviations

PM: peritoneal metastasis
GC: gastric cancer
BMDC: bone marrow-derived cells
EMT: epithelial-mesenchymal transformation
HOXA11: homeobox a11
HPMC: human peritoneal mesothelial cell
MMT: mesenchymal transition
EGR1: early growth response 1
PN-1: protease connexin-1
CAFs: cancer-associated fibroblasts
NIPS: neoadjuvant intraperitoneal and systemic chemotherapy
PCD: positive cell density
KEGG: Kyoto Encyclopedia of Genes and Genomes
CD44s: standard CD44
CD44v: variant CD44
CSCs: cancer stem cells
