# Supplementary material for: Blocking EGR1/TGF-β1 and CD44s/STAT3 Crosstalk Inhibits Peritoneal Metastasis of Gastric Cancer
Source: Int J Biol Sci. 2024 Jan 27;20(4):1314–31. doi: 10.7150/ijbs.90598 (PMC10878142; doi:10.7150/ijbs.90598)
Supplement: Supplementary file 1 — Supplementary figures and tables. [file ijbsv20p1314s1.pdf]

Supplementary tables

Table S1. Antibodies used in this study

Antibody	catalog	Dilution	Company
**For Western blotting**			
EGR1	22008-1-AP	1:1000	Proteintech
N-Cadherin (D4R1H)	13116	1:1000	CST
E-Cadherin	A16811	1:1000	Abclone
CD44 (156-3C11)	3570S	1:1000	CST
Vimentin (D21H3)	5741	1:1000	CST
α-SMA	ab5694	1:500	Abcam
Bmi1 (D20B7)	6964T	1:500	CST
Nanog (D73G4)	4903	1:800	CST
Twist1	25465-1-AP	1:500	Proteintech
TGF-β1 (56E4)	3709	1:1000	CST
STAT3	10253-2-AP	1:1000	Proteintech
P-STAT3	ARE6058	1:1000	AR antibody Revolution
GAPDH (D16H11)	5174S	1:1000	CST
Secondary antibody	7076S	1:5000	CST
Secondary antibody	7074S	1:5000	CST
**For Immunohistochemistry**			
EGR1	22008-1-AP	1:300	Proteintech
Bmi1 (D20B7)	6964T	1:200	CST
Nanog (D73G4)	4903	1:400	CST
CD44	15675-1-AP	1:500	Proteintech
P-STAT3	ARE6058	1:100	AR antibody Revolution
Secondary antibody	Envision kit (HRP, rabbit/mouse, DAB+)	Ready-to-use	DAKO

For Immunofluorescence staining			
EGR1	22008-1-AP	1:300	Proteintech
HBME1	M3505	1:50	DAKO
HOXA11	Ab54365	1:250	Abcam
CD44	15675-1-AP	1:500	Proteintech
Ki67	GM724029	1:50	Gene Tech
TGF-β1	TA1027	1:50	Abmart
STAT3	10253-2-AP	1:200	Proteintech
P-STAT3	9145S	1:100	CST
DAPI	C1002	1:1000	Beyotime
Secondary antibody	Alexa Fluor 594 anti-mouse IgG	1:50	Invitrogen
Secondary antibody	Alexa Fluor 594 anti-rabbit IgG	1:50	Invitrogen
Secondary antibody	Alexa Fluor 488 anti-rabbit IgG	1:50	Invitrogen
Secondary antibody	Alexa Fluor 647 anti-mouse IgG	1:50	Invitrogen
Secondary antibody	Alexa Fluor 680 anti-rabbit IgG	1:50	Invitrogen

Table S2. The sequences of gene-specific primers used for qRT-PCR, vector constructs and ChIP assay

Gene name	Forward (5'-3')	Reverse (5'-3')
**Primers for qRT-PCR**		
EGR1	GGTCAGTGGCCTAGTGAGC	GTGCCGCTGAGTAAATGGGA
CD44s	AATCCCTGCTACCAGAGACC	TTCAGATCCATGAGTGGTATGGG
CD44v5-v8	TCCCTGCTACCAATATGGACTC	CAGAGTAGAAGTTGTTGGATGGTC
CD44v2	GCAACCAAGAGGCAAGAAA	CAGCCATTTGTGTTGTTGTGTG
CD44v3	CGTCTTCAAATACCATCTCAGC	CAATGCCTGATCCAGAAAAAC
CD44v4	TGACCACACAAAACAGAACC	GTTGTCTGAAGTAGCACTTCC
CD44v5	GAAATGGCACCACTGCTTATG	GTCTCTTCTTCCTCATGATGCT
CD44v6	AGGAACAGTGGTTTGGCAAC	CGAATGGGAGTCTTCTCTGG
CD44v7	TCAGCTCATACCAGCCATCC	TCCTTCTTCCTGCTTGATGAC
CD44v8	TCAGCCTACTGCAAATCCAA	GAGGTCCTGTCCTGTCCAAA
CD44v9	AGCAGAGTAATTCTCAGAGCTTC	TCAGAGTAGAAGTTGTTGGATGG
CD44v10	GGAATGATGTCACAGGTGGA	AGGTCACTGGGATGAAGGTC
**Primers for vector constructs**		
H_TGFβ1 promoter(-1900 to +100) WT	AGGTACCGAGCTCTTACGCGTC CCACCCTCACCCTACCCA	CTTACTTAGATCGCAGATCTCGAGG GGGCGTCCCCCCTGCCC
H_TGFβ1 promoter(-1900 to -183) WT	AGGTACCGAGCTCTTACGCGTC CCACCCTCACCCTACCCA	CTTACTTAGATCGCAGATCTCGAGC CCCGGCTCCGCCCCGCAA
H_TGFβ1 promoter(-1900 to +100) MT1	AGGTACCGAGCTCTTACGCGTC CCACCCTCACCCTACCCA	CTTACTTAGATCGCAGATCTCGAGG GGGCGTCCCCCCTGCCC
H_TGFβ1 promoter(-1900 to +100) MT2	AGGTACCGAGCTCTTACGCGTC CCACCCTCACCCTACCCA	CTTACTTAGATCGCAGATCTCGAGG GGGCGTCCCCCCTGCCC
H_EGR1	GCGAATTCGAAGTATACCTCGAG GCCACCATGGCCGCGGCCA	CGATCGCAGATCCTTGGATCCTTAG CAAATTTCAATTGTCCTGGGAG
**Primers for ChIP**		
TGF-β1	ATTAAGCCTTCTCCGCCTGGT	TCGGCAGGGGGTTTTGAAGCC

Supplementary figures and legends

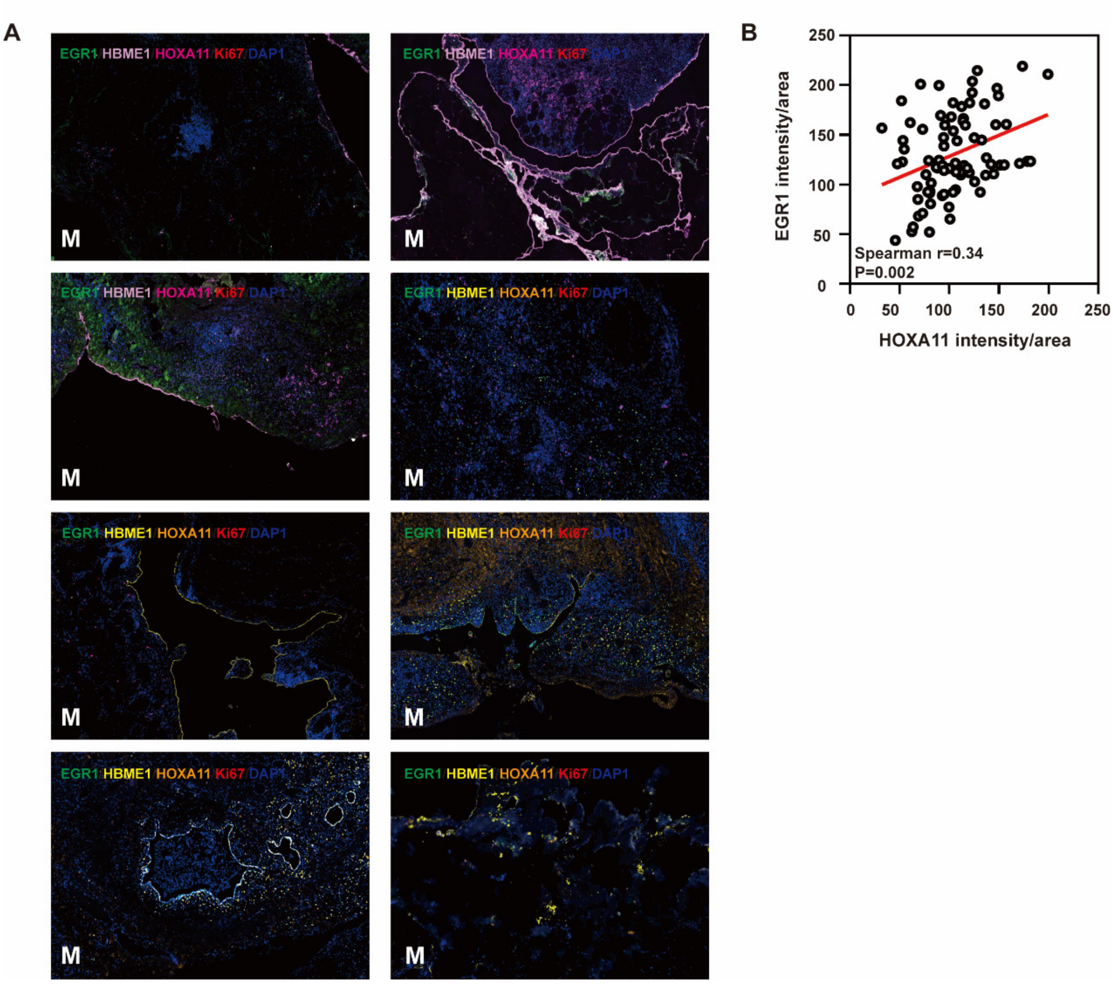

Figure S1. A. Multiple immunofluorescences staining of the PM and the matched paracancerous tissue in eight patients with GC confirmed by pathology (M: peritoneal metastasis) (n=8). B. Spearman correlation analysis of mean fluorescence intensity between HOXA11 and EGR1.

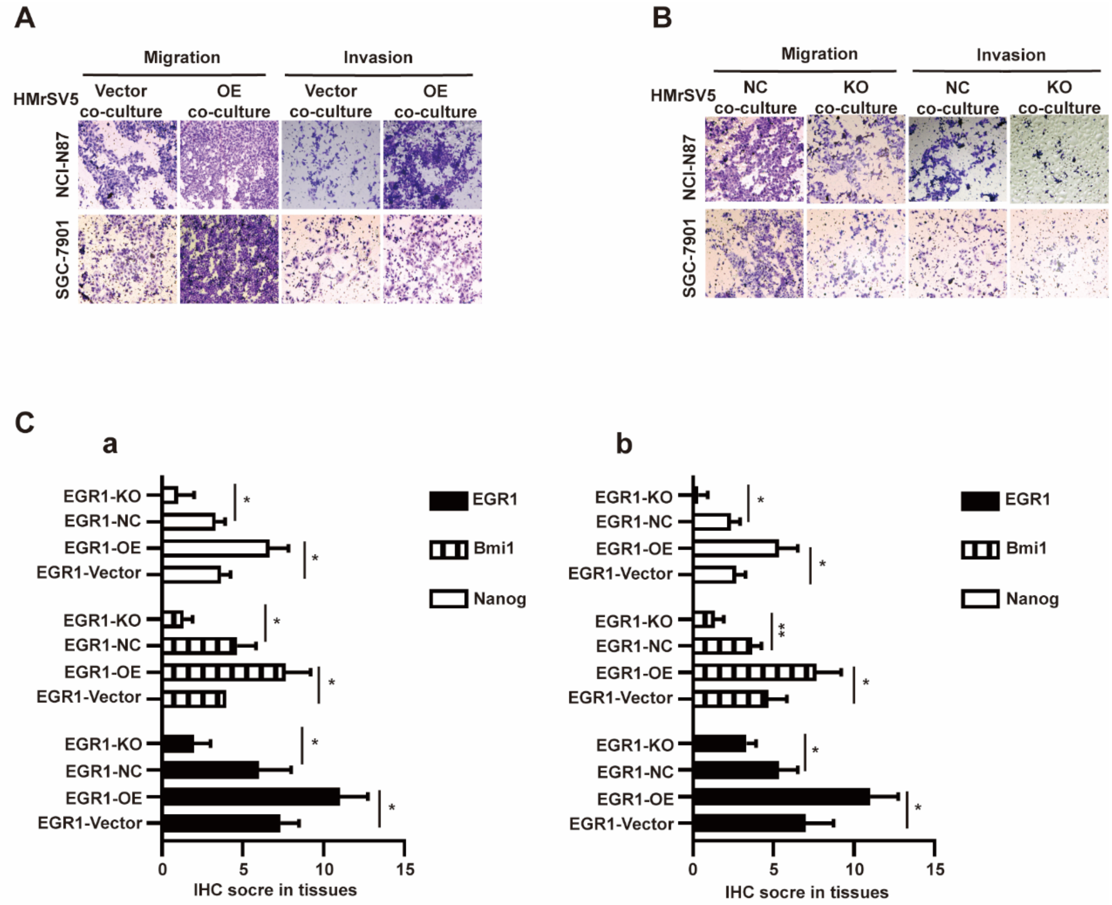

Figure S2. A-B. Cell migration and invasion were analyzed in NCI-N87 and SGC-7901 co-cultured with EGR1-overexpressing HMrSV5 cells (A) and with EGR1-knockout HMrSV5 cells (B). Ca-b. The statistical analyses of EGR1, Bmi1, and Nanog expression were evaluated by IHC score in tissues of xenografts. *, $P < 0.05$; **, $P < 0.01$.

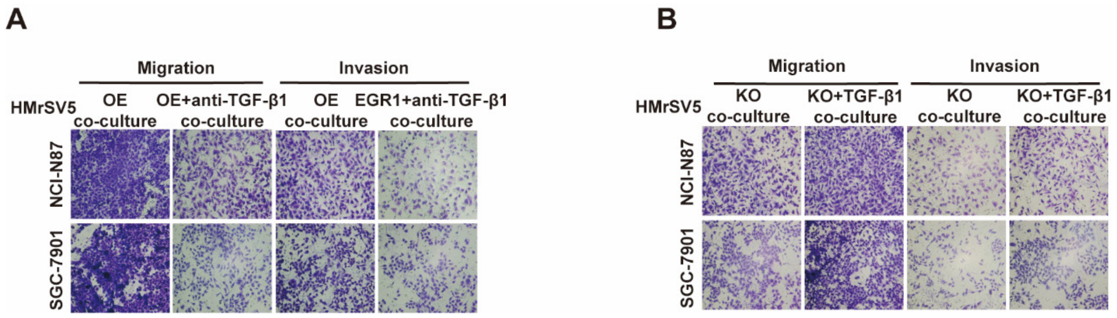

Figure S3. A-B. Cell migration and invasion were analyzed in NCI-N87 and SGC-7901 treated with TGF-β1 neutralizing antibody in EGR1-overexpressing HMrSV5 cells co-cultured system (A) and with recombinant TGF-β1 in EGR1-knockout HMrSV5 cells co-cultured system (B).

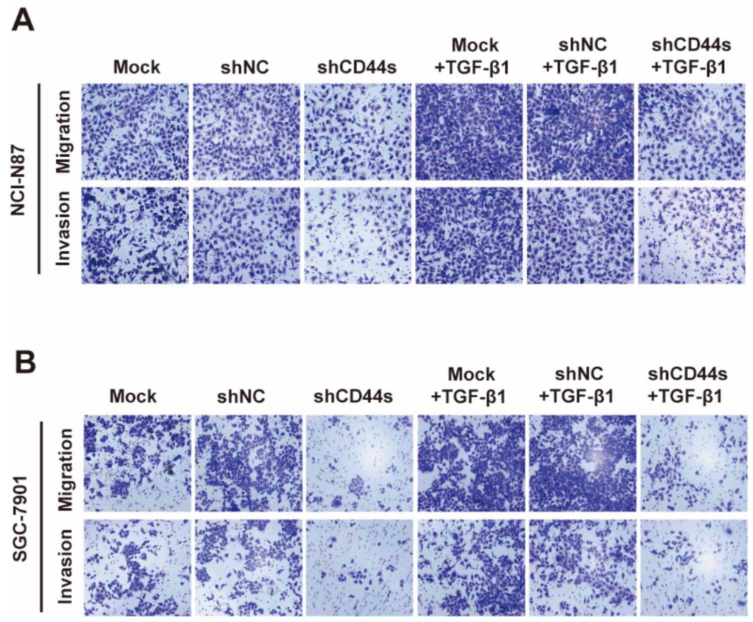

Figure S4. A-B. Cell migration and invasion were analyzed in CD44s-konckdown NCI-N87 (A) and SGC-7901 (B) cells and their respective control groups co-cultured in presence or absence of TGF-β1.

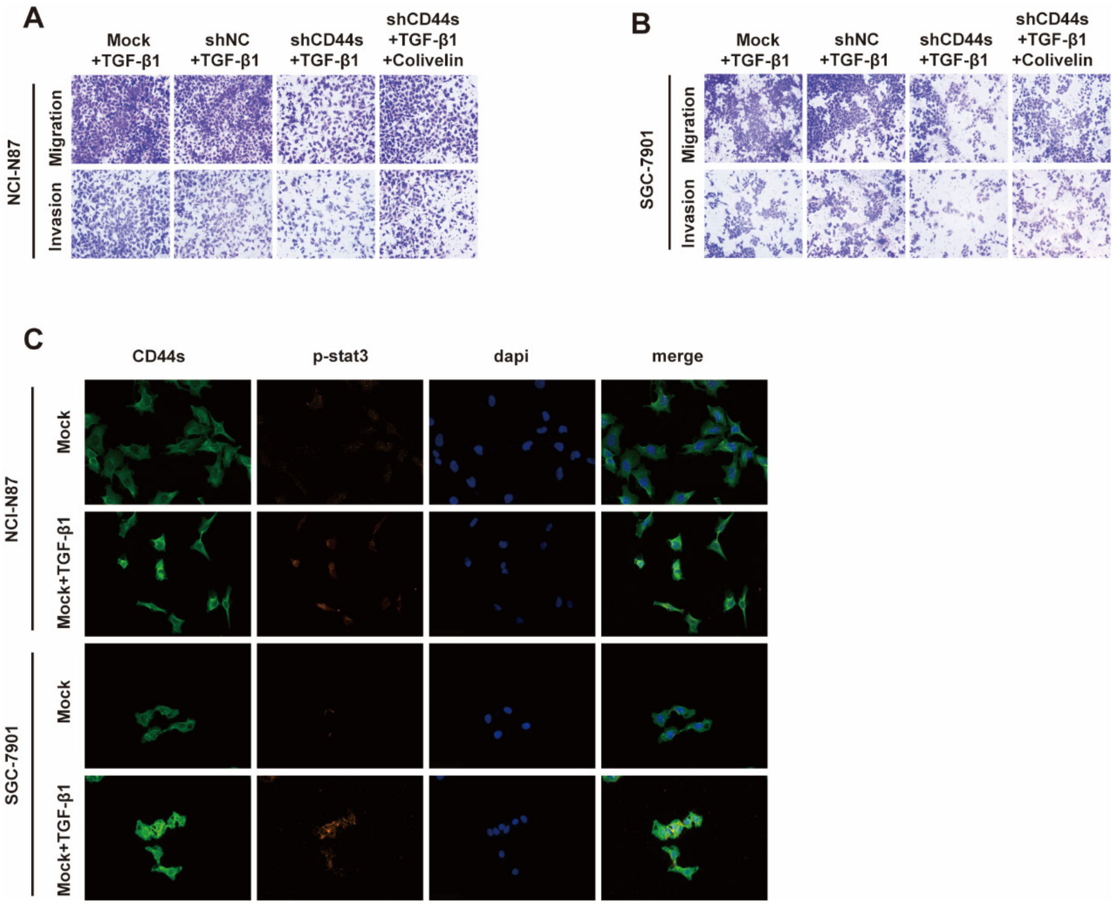

Figure S5. A-B. Cell migration and invasion were analyzed in CD44s-konckdown NCI-N87 (A) and SGC-7901 (B) cells and their respective control groups treated in presence or absence of Colivelin in TGF-β1 co-culture system. C. Immunofluorescence assay evaluating the cell internalization of CD44s and its interaction with p-STAT3 in NCI-N87 and SGC-7901 cells in the presence or absence of TGF-β1 stimulation.

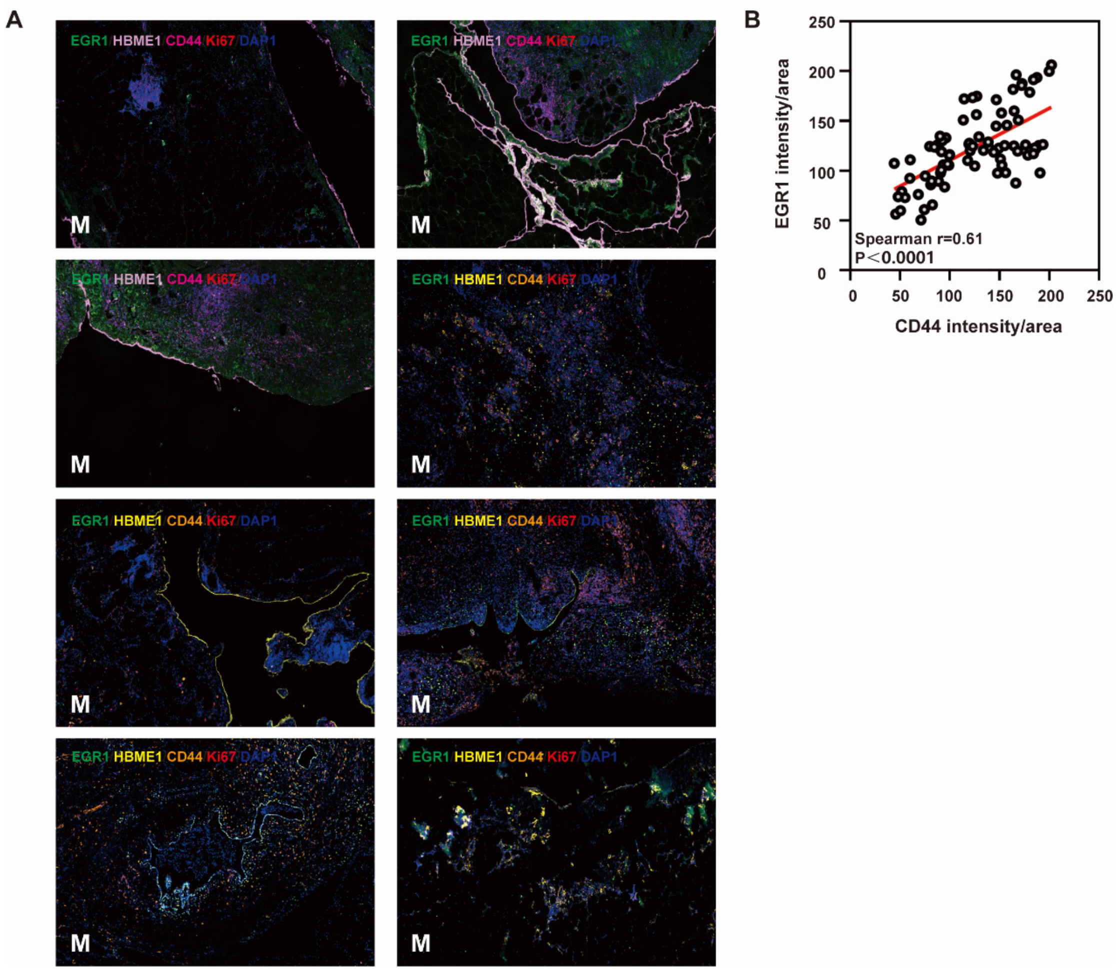

Figure S6. A. Multiple immunofluorescences staining of the PM and the matched paracancerous tissue in eight patients with GC confirmed by pathology (M: peritoneal metastasis) (n=8). B. Spearman correlation analysis of mean fluorescence intensity between CD44 and EGR1.

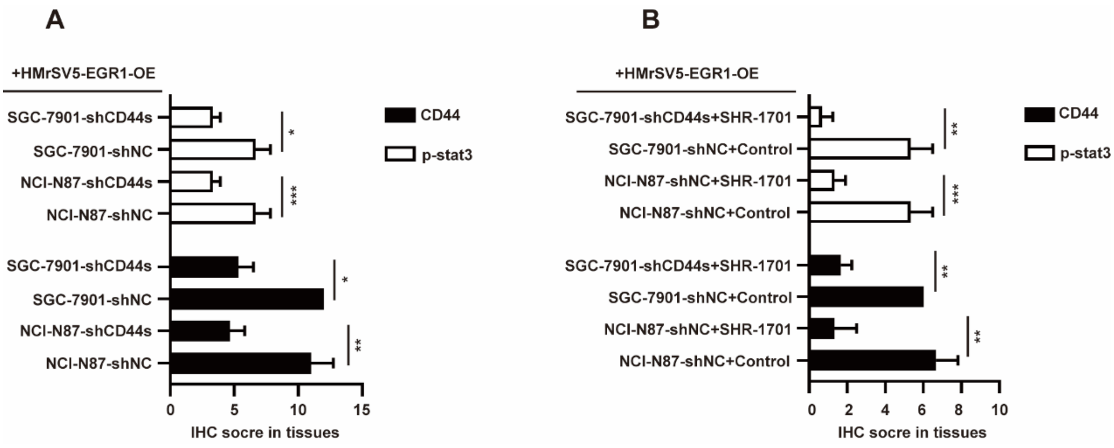

Figure S7. A-B. The statistical analyses of CD44 and p-STAT3 expression were evaluated by IHC score in tissues of xenografts. *, P < 0.05; **, P < 0.01; ***, P < 0.001.

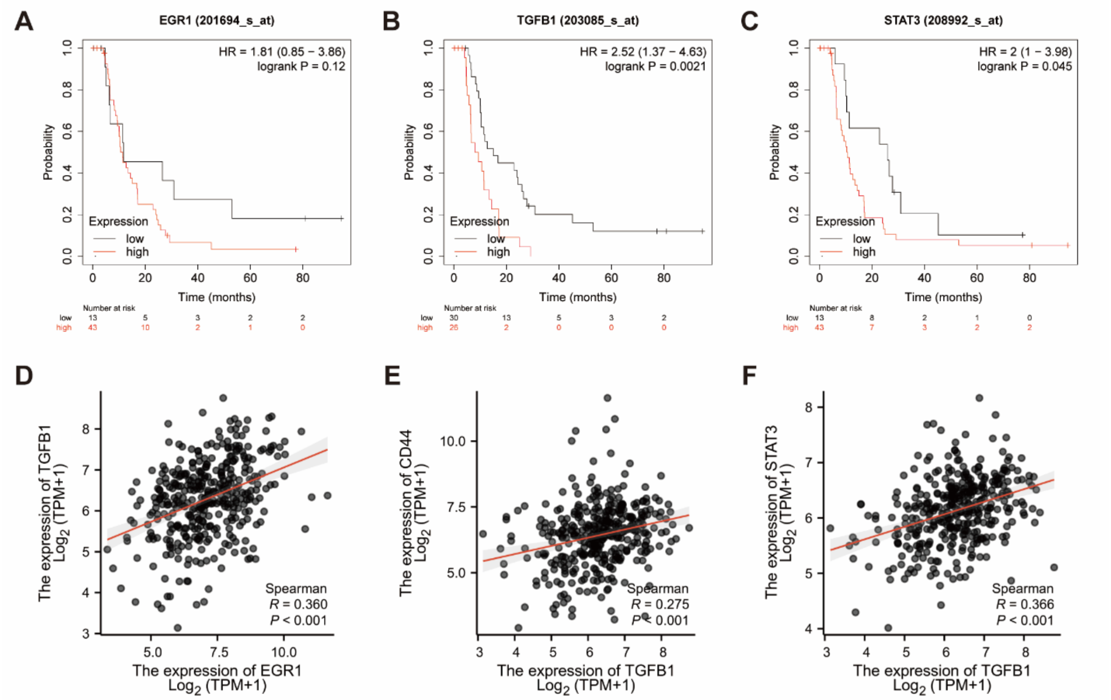

Figure S8. A-C. Kaplan-Meier survival analysis between the low and high expression groups of EGR1, TGFB1 and STAT3 in M1 stage GC tissues. D-F. Correlation analysis among EGR1, TGFB1, CD44 and STAT3 in human GC tissues based on TCGA database.
